# Supplementary material for: One-Dimensional Mercury Halide Coordination Polymers Based on A Semi-Rigid N-Donor Ligand: Reversible Structural Transformation
Source: Polymers (Basel). 2019 Mar 6;11(3):436. doi: 10.3390/polym11030436 (PMC6473486; doi:10.3390/polym11030436)

checkCIF/PLATON report

Structure factors have been supplied for datablock(s) 1, 2, 3, 4

THIS REPORT IS FOR GUIDANCE ONLY. IF USED AS PART OF A REVIEW PROCEDURE FOR PUBLICATION, IT SHOULD NOT REPLACE THE EXPERTISE OF AN EXPERIENCED CRYSTALLOGRAPHIC REFEREE.

No syntax errors found. CIF dictionary Interpreting this report

Datablock: 1

Bond precision: C-C = 0.0066 A Wavelength=0.71073

Cell:	a=5.0705(1)	b=20.6636(5)	c=11.4320(3)
	alpha=90	beta=92.3088(18)	gamma=90
Temperature:	296 K		

	Calculated	Reported
Volume	1196.81(5)	1196.81(5)
Space group	P 21/m	P 21/m
Hall group	-P 2yb	-P 2yb
Moiety formula	C20 H18 Cl2 Hg N4 O2, C H4 O	?
Sum formula	C21 H22 Cl2 Hg N4 O3	C21 H22 Cl2 Hg N4 O3
Mr	649.92	649.91
Dx,g cm-3	1.804	1.803
Z	2	2
Mu (mm-1)	6.682	6.682
F000	628.0	628.0
F000’	624.19	
h,k,lmax	6,27,15	6,27,15
Nref	3077	3057
Tmin,Tmax	0.453,0.716	0.480,0.746
Tmin’	0.133	

Correction method= # Reported T Limits: Tmin=0.480 Tmax=0.746
AbsCorr = MULTI_SCAN

Data completeness= 0.994 Theta(max)= 28.369

R(reflections)= 0.0364(2395) wR2(reflections)= 0.0783(3057)

S = 1.009 Npar= 143

```
The following ALERTS were generated. Each ALERT has the format
       test-name_ALERT_alert-type_alert-level.
Click on the hyperlinks for more details of the test.
```

Alert level C

```
PLAT202_ALERT_3_C Isotropic non-H Atoms in Anion/Solvent .........          1 Check
PLAT241_ALERT_2_C High   'MainMol' Ueq as Compared to Neighbors of         C7 Check
PLAT242_ALERT_2_C Low    'MainMol' Ueq as Compared to Neighbors of         C6 Check
PLAT911_ALERT_3_C Missing FCF Refl Between Thmin & STh/L=    0.600          5 Report
```

Alert level G

```
PLAT002_ALERT_2_G Number of Distance or Angle Restraints on AtSite          2 Note
PLAT004_ALERT_5_G Polymeric Structure Found with Maximum Dimension          1 Info
PLAT007_ALERT_5_G Number of Unrefined Donor-H Atoms ..............          3 Report
PLAT172_ALERT_4_G The CIF-Embedded .res File Contains DFIX Records          1 Report
PLAT300_ALERT_4_G Atom Site Occupancy of O2         Constrained at        0.5 Check
PLAT300_ALERT_4_G Atom Site Occupancy of H11A       Constrained at        0.5 Check
PLAT300_ALERT_4_G Atom Site Occupancy of H11B       Constrained at        0.5 Check
PLAT300_ALERT_4_G Atom Site Occupancy of H2C        Constrained at       0.25 Check
PLAT300_ALERT_4_G Atom Site Occupancy of H2D        Constrained at       0.25 Check
PLAT302_ALERT_4_G Anion/Solvent/Minor-Residue Disorder (Resd  2  )        50% Note
PLAT764_ALERT_4_G Overcomplete CIF Bond List Detected (Rep/Expd) .       1.16 Ratio
PLAT860_ALERT_3_G Number of Least-Squares Restraints .............          1 Note
PLAT910_ALERT_3_G Missing # of FCF Reflection(s) Below Theta(Min).          2 Note
PLAT912_ALERT_4_G Missing # of FCF Reflections Above STh/L=  0.600         14 Note
PLAT933_ALERT_2_G Number of OMIT Records in Embedded .res File ...          4 Note
PLAT978_ALERT_2_G Number C-C Bonds with Positive Residual Density.          1 Info
```

```
   0 ALERT level A = Most likely a serious problem - resolve or explain
   0 ALERT level B = A potentially serious problem, consider carefully
   4 ALERT level C = Check. Ensure it is not caused by an omission or oversight
  16 ALERT level G = General information/check it is not something unexpected

   0 ALERT type 1 CIF construction/syntax error, inconsistent or missing data
   5 ALERT type 2 Indicator that the structure model may be wrong or deficient
   4 ALERT type 3 Indicator that the structure quality may be low
   9 ALERT type 4 Improvement, methodology, query or suggestion
   2 ALERT type 5 Informative message, check
```

Datablock: 2

```
Bond precision:  C-C = 0.0044 A          Wavelength=0.71073

Cell:            a=9.3764(5)    b=13.3232(7)      c=17.1229(10)
                 alpha=90       beta=97.490(2)    gamma=90
Temperature:     299 K
```

	Calculated	Reported
Volume	2120.8(2)	2120.8(2)
Space group	P 21/c	P 21/c
Hall group	-P 2ybc	-P 2ybc
Moiety formula	C20 H18 Cl2 Hg N4 O2	?
Sum formula	C20 H18 Cl2 Hg N4 O2	C20 H18 Cl2 Hg N4 O2
Mr	617.87	617.87
Dx,g cm-3	1.935	1.935
Z	4	4
Mu (mm-1)	7.533	7.533
F000	1184.0	1184.0
F000'	1176.38	
h,k,lmax	12,17,22	12,17,22
Nref	5284	5275
Tmin,Tmax	0.179,0.471	0.547,0.746
Tmin'	0.096	

Correction method= # Reported T Limits: Tmin=0.547 Tmax=0.746
AbsCorr = MULTI_SCAN

Data completeness= 0.998 Theta(max)= 28.311

R(reflections)= 0.0255(4223) wR2(reflections)= 0.0524(5275)

S = 1.032 Npar= 262

The following ALERTS were generated. Each ALERT has the format
test-name_ALERT_alert-type_alert-level.
Click on the hyperlinks for more details of the test.

Alert level C

PLAT241_ALERT_2_C High 'MainMol' Ueq as Compared to Neighbors of C14 Check

---

Alert level G

PLAT004_ALERT_5_G Polymeric Structure Found with Maximum Dimension 1 Info
PLAT007_ALERT_5_G Number of Unrefined Donor-H Atoms .... 2 Report
PLAT232_ALERT_2_G Hirshfeld Test Diff (M-X) Hg --N1 . 5.0 s.u.
PLAT804_ALERT_5_G Number of ARU-Code Packing Problem(s) in PLATON 1 Info
PLAT912_ALERT_4_G Missing # of FCF Reflections Above STh/L= 0.600 10 Note
PLAT978_ALERT_2_G Number C-C Bonds with Positive Residual Density. 1 Info

---

0 **ALERT level A** = Most likely a serious problem - resolve or explain
0 **ALERT level B** = A potentially serious problem, consider carefully
1 **ALERT level C** = Check. Ensure it is not caused by an omission or oversight
6 **ALERT level G** = General information/check it is not something unexpected

0 ALERT type 1 CIF construction/syntax error, inconsistent or missing data

3 ALERT type 2 Indicator that the structure model may be wrong or deficient
0 ALERT type 3 Indicator that the structure quality may be low
1 ALERT type 4 Improvement, methodology, query or suggestion
3 ALERT type 5 Informative message, check

Datablock: 3

Bond precision: C-C = 0.0058 A   Wavelength=0.71073

Cell:	a=9.3217(1)	b=13.4660(1)	c=17.2458(2)
	alpha=90	beta=96.741(1)	gamma=90
Temperature:	296 K		

	Calculated	Reported
Volume	2149.83(4)	2149.83(4)
Space group	P 21/c	P 21/c
Hall group	-P 2ybc	-P 2ybc
Moiety formula	C20 H18 Br2 Hg N4 O2	?
Sum formula	C20 H18 Br2 Hg N4 O2	C20 H18 Br2 Hg N4 O2
Mr	706.77	706.79
Dx,g cm-3	2.184	2.184
Z	4	4
Mu (mm-1)	10.900	10.900
F000	1328.0	1328.0
F000’	1317.41	
h,k,lmax	12,17,22	12,17,22
Nref	5339	5335
Tmin,Tmax	0.281,0.336	0.513,0.746
Tmin’	0.037	

Correction method= # Reported T Limits: Tmin=0.513 Tmax=0.746
AbsCorr = MULTI_SCAN

Data completeness= 0.999   Theta(max)= 28.298

R(reflections)= 0.0312(3982)   wR2(reflections)= 0.0608(5335)

S = 1.023   Npar= 262

The following ALERTS were generated. Each ALERT has the format
test-name_ALERT_alert-type_alert-level.
Click on the hyperlinks for more details of the test.

Alert level C

PLAT241_ALERT_2_C High ’MainMol’ Ueq as Compared to Neighbors of C14 Check
PLAT978_ALERT_2_C Number C-C Bonds with Positive Residual Density. 0 Info

---

Alert level G

```
PLAT004_ALERT_5_G Polymeric Structure Found with Maximum Dimension          1 Info
PLAT007_ALERT_5_G Number of Unrefined Donor-H Atoms ..............          2 Report
PLAT142_ALERT_4_G s.u. on b - Axis Small or Missing ..............    0.00010 Ang.
PLAT232_ALERT_2_G Hirshfeld Test Diff (M-X)  Hg       --Br1      .        8.0 s.u.
PLAT912_ALERT_4_G Missing # of FCF Reflections Above STh/L=  0.600          5 Note
```

---

0 **ALERT level A** = Most likely a serious problem - resolve or explain
0 **ALERT level B** = A potentially serious problem, consider carefully
2 **ALERT level C** = Check. Ensure it is not caused by an omission or oversight
5 **ALERT level G** = General information/check it is not something unexpected

0 ALERT type 1 CIF construction/syntax error, inconsistent or missing data
3 ALERT type 2 Indicator that the structure model may be wrong or deficient
0 ALERT type 3 Indicator that the structure quality may be low
2 ALERT type 4 Improvement, methodology, query or suggestion
2 ALERT type 5 Informative message, check

Datablock: 4

```
Bond precision:  C-C = 0.0081 A          Wavelength=0.71073

Cell:             a=9.3679(2)    b=13.6718(3)      c=17.3825(5)
                  alpha=90       beta=95.555(1)    gamma=90
Temperature:      296 K

                 Calculated                 Reported
Volume           2215.83(9)                 2215.83(9)
Space group      P 21/c                     P 21/c
Hall group       -P 2ybc                    -P 2ybc
Moiety formula   C20 H18 Hg I2 N4 O2        ?
Sum formula      C20 H18 Hg I2 N4 O2        C20 H18 Hg I2 N4 O2
Mr               800.77                     800.77
Dx,g cm-3        2.400                      2.400
Z                4                          4
Mu (mm-1)        9.756                      9.756
F000             1472.0                     1472.0
F000'            1459.68
h,k,lmax         12,18,23                   12,18,23
Nref             5535                       5516
Tmin,Tmax        0.323,0.614                0.296,0.746
Tmin'            0.019

Correction method= # Reported T Limits: Tmin=0.296 Tmax=0.746
AbsCorr = MULTI_SCAN

Data completeness= 0.997          Theta(max)= 28.336
```

```
R(reflections)= 0.0337( 4779)     wR2(reflections)= 0.0882( 5516)

S = 1.022                    Npar= 262
```

The following ALERTS were generated. Each ALERT has the format
test-name_ALERT_alert-type_alert-level.
Click on the hyperlinks for more details of the test.

Alert level C

```
PLAT342_ALERT_3_C Low Bond Precision on  C-C Bonds ...............    0.00806 Ang.
PLAT420_ALERT_2_C D-H Without Acceptor       N3       --H3A      .     Please Check
PLAT906_ALERT_3_C Large K Value in the Analysis of Variance ......      2.271 Check
PLAT971_ALERT_2_C Check Calcd Resid. Dens.  0.76A   From Hg              1.97 eA-3
PLAT972_ALERT_2_C Check Calcd Resid. Dens.  0.87A   From Hg             -1.65 eA-3
PLAT978_ALERT_2_C Number C-C Bonds with Positive Residual Density.          0 Info
```

Alert level G

```
PLAT004_ALERT_5_G Polymeric Structure Found with Maximum Dimension          1 Info
PLAT007_ALERT_5_G Number of Unrefined Donor-H Atoms .............           2 Report
PLAT083_ALERT_2_G SHELXL Second Parameter in WGHT  Unusually Large      10.32 Why ?
PLAT232_ALERT_2_G Hirshfeld Test Diff (M-X)  Hg       --I1       .        9.0 s.u.
PLAT912_ALERT_4_G Missing # of FCF Reflections Above STh/L=  0.600         19 Note
```

---

0 **ALERT level A** = Most likely a serious problem - resolve or explain
0 **ALERT level B** = A potentially serious problem, consider carefully
6 **ALERT level C** = Check. Ensure it is not caused by an omission or oversight
5 **ALERT level G** = General information/check it is not something unexpected

0 ALERT type 1 CIF construction/syntax error, inconsistent or missing data
6 ALERT type 2 Indicator that the structure model may be wrong or deficient
2 ALERT type 3 Indicator that the structure quality may be low
1 ALERT type 4 Improvement, methodology, query or suggestion
2 ALERT type 5 Informative message, check

It is advisable to attempt to resolve as many as possible of the alerts in all categories. Often the minor alerts point to easily fixed oversights, errors and omissions in your CIF or refinement strategy, so attention to these fine details can be worthwhile. In order to resolve some of the more serious problems it may be necessary to carry out additional measurements or structure refinements. However, the purpose of your study may justify the reported deviations and the more serious of these should normally be commented upon in the discussion or experimental section of a paper or in the "special_details" fields of the CIF. checkCIF was carefully designed to identify outliers and unusual parameters, but every test has its limitations and alerts that are not important in a particular case may appear. Conversely, the absence of alerts does not guarantee there are no aspects of the results needing attention. It is up to the individual to critically assess their own results and, if necessary, seek expert advice.

Publication of your CIF in IUCr journals

A basic structural check has been run on your CIF. These basic checks will be run on all CIFs submitted for publication in IUCr journals (*Acta Crystallographica*, *Journal of Applied Crystallography*, *Journal of Synchrotron Radiation*); however, if you intend to submit to *Acta Crystallographica Section C* or *E* or *IUCrData*, you should make sure that full publication checks are run on the final version of your CIF prior to submission.

Publication of your CIF in other journals

Please refer to the *Notes for Authors* of the relevant journal for any special instructions relating to CIF submission.

PLATON version of 06/01/2019; check.def file version of 19/12/2018

Datablock 1 - ellipsoid plot

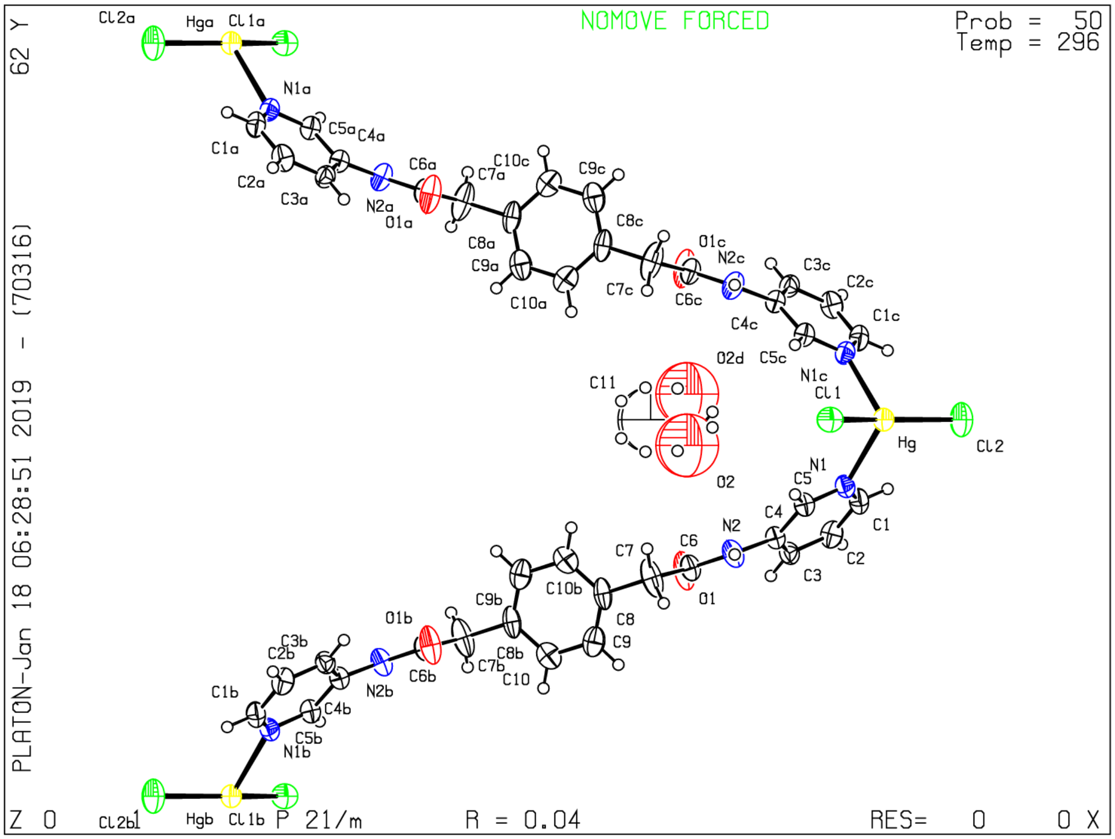

Datablock 2 - ellipsoid plot

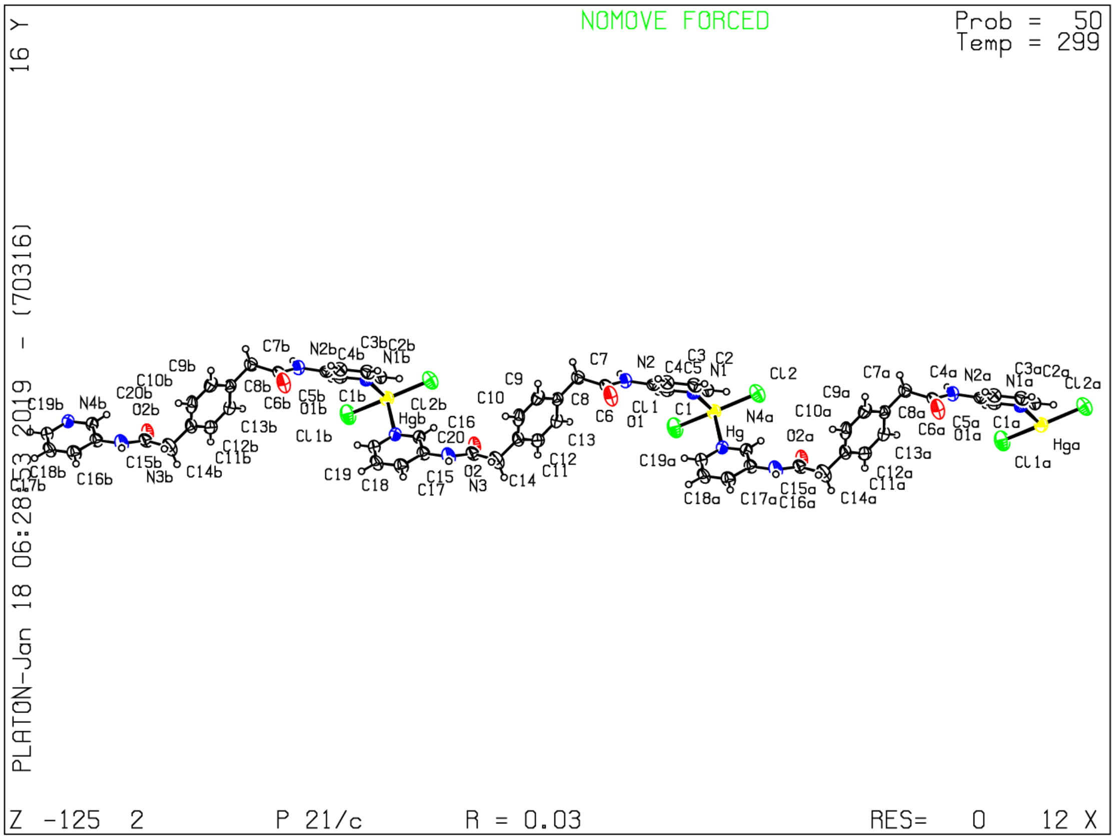

Datablock 3 - ellipsoid plot

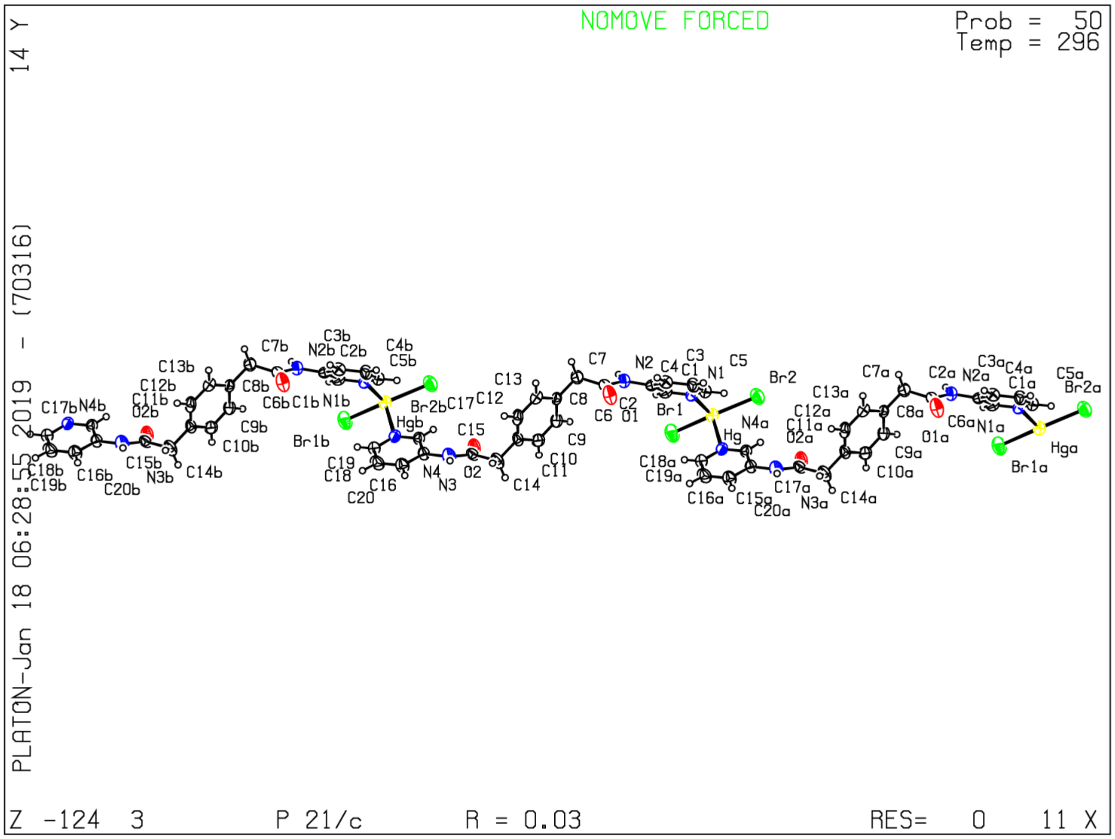

Datablock 4 - ellipsoid plot

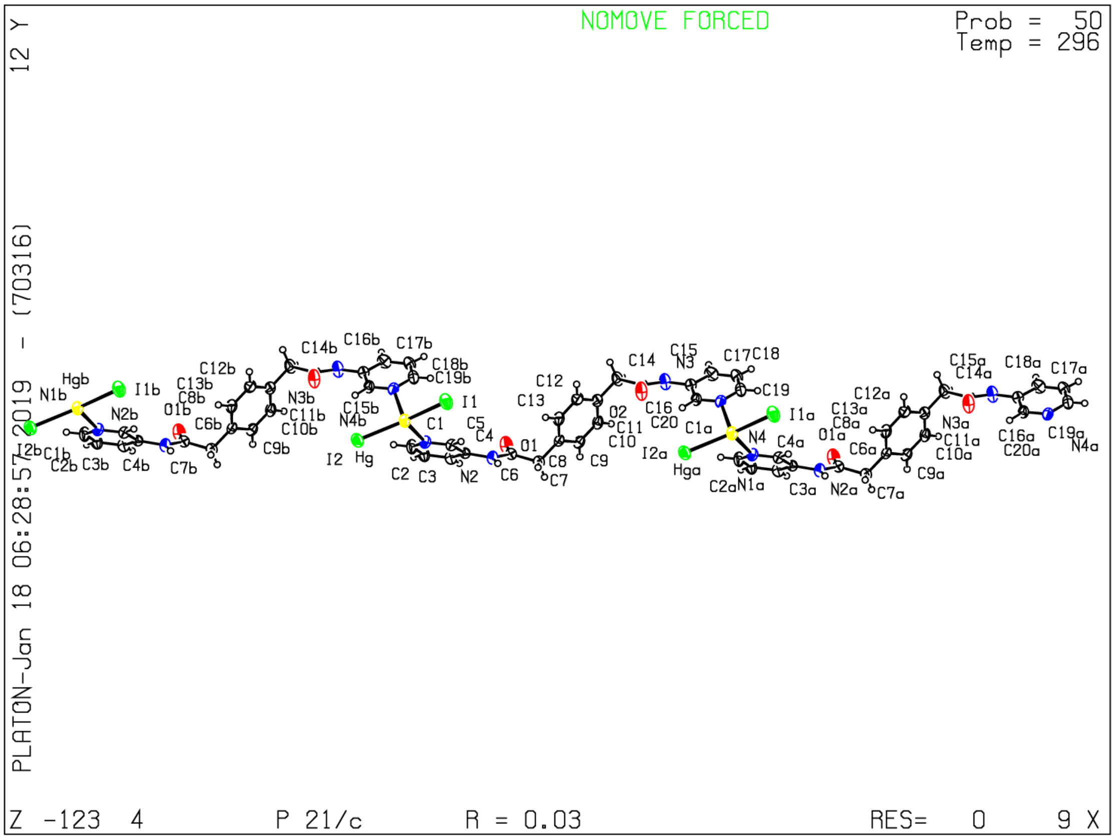

Supplement: Supplementary file 1 [file polymers-11-00436-s001.zip › polymers-455907-SP-English/polymers-455907-supplementary/checkcif.pdf]
