# Supplementary material for: One-Dimensional Mercury Halide Coordination Polymers Based on A Semi-Rigid N-Donor Ligand: Reversible Structural Transformation
Source: Polymers (Basel). 2019 Mar 6;11(3):436. doi: 10.3390/polym11030436 (PMC6473486; doi:10.3390/polym11030436)
Supplement: Supplementary file 1 [file polymers-11-00436-s001.zip › polymers-455907-SP-English/polymers-455907-supplementary/Polymer_SI-1.docx]

##### One-Dimensional Mercury Halide Coordination Polymers Based on a Semi-Rigid N-Donor Ligand: Reversible Structural Transformation

Pradhumna Mahat Chhetri ^1,2^, Xiang-Kai Yang^1^, Chih-Tung Yang^1^ and Jhy-Der Chen^1^*

*1. Department of Chemistry, Chung-Yuan Christian University, Chung-Li, Taiwan, R.O.C.*

*2. Department of Chemistry, Amrit Science Campus, Tribhuvan University, Kathmandu, Nepal.*

**Figure S1.** Powder X-ray patterns of **1**. (**a**) Simulation and (**b**) experimental.


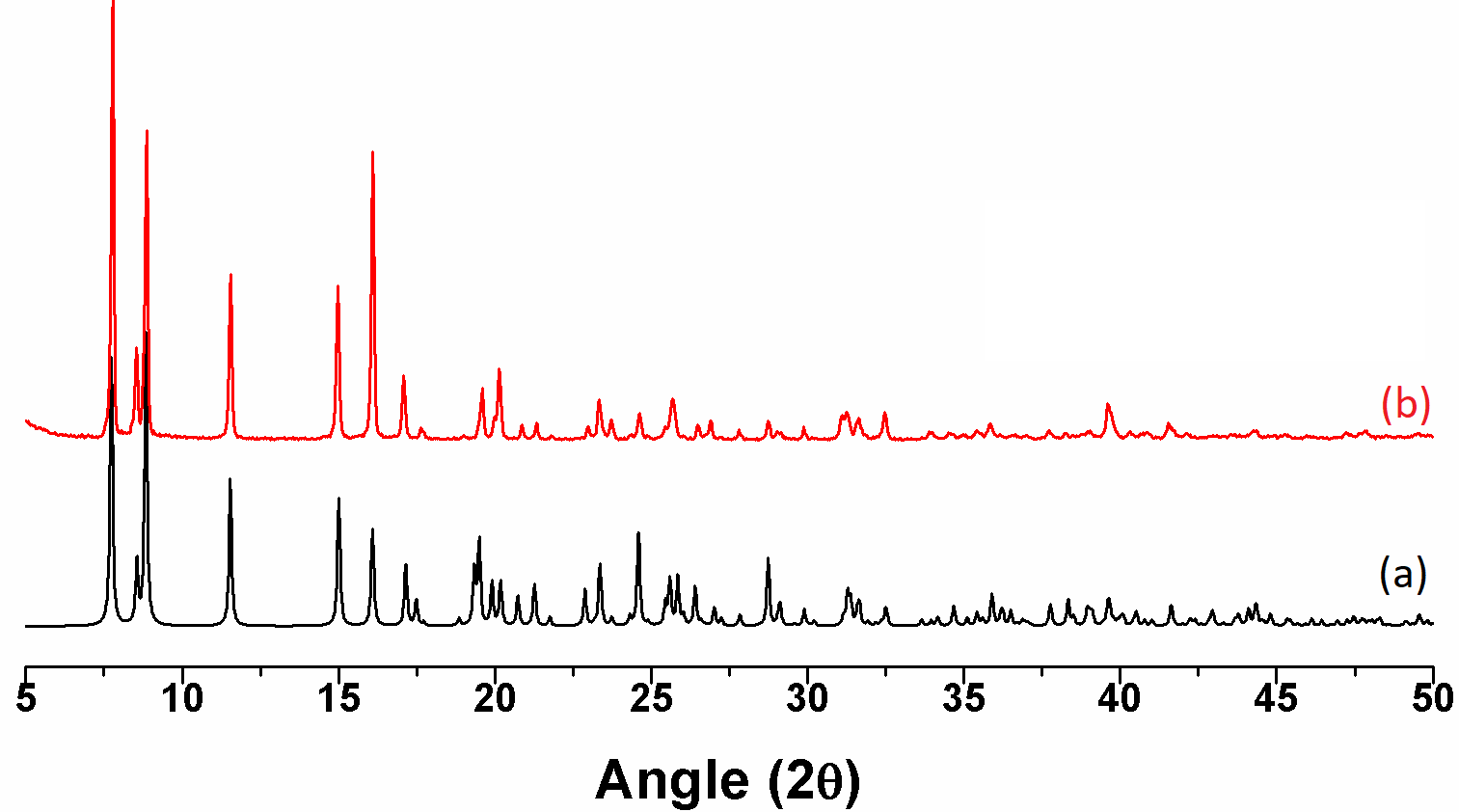


**Figure S2.** Powder X-ray patterns of **2**. (**a**) simulation and (**b**) experimental.


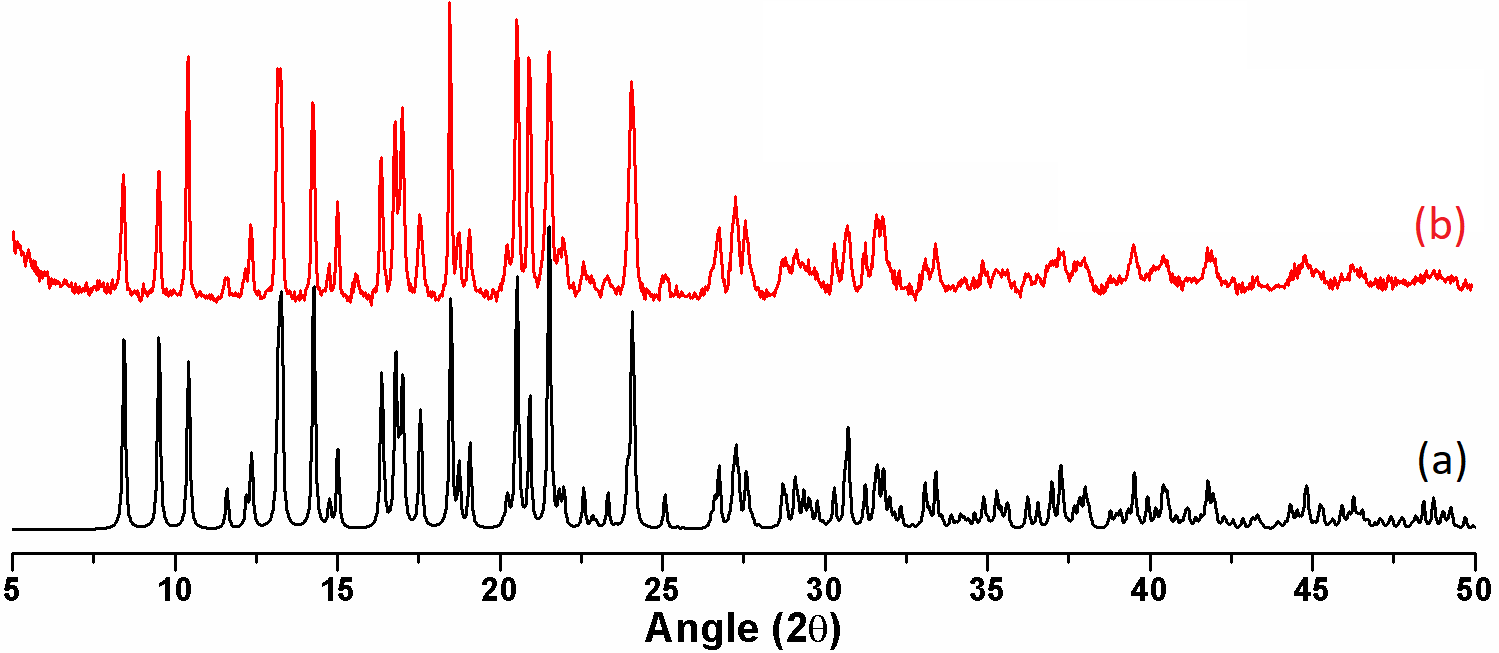


**Figure S3.** Powder X-ray patterns of **3**. (**a**) Simulation, (**b**) experimental of **3** obtained from THF/MeOH, and (**c**) experimental of **3** obtained from THF/EtOH.


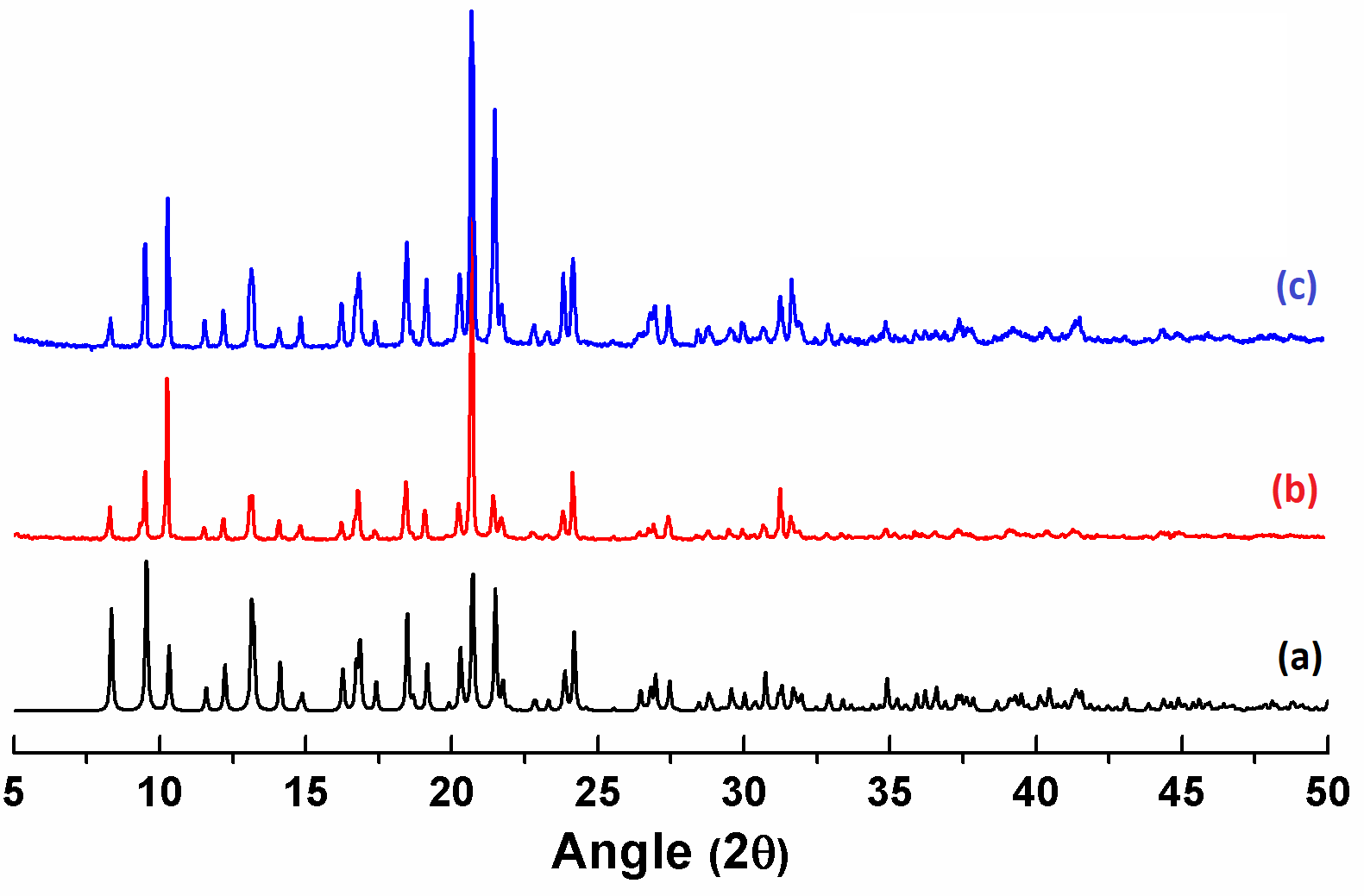


**Figure S4.** Powder X-ray patterns of **4**. (**a**) $simulation, (**b**) experimental of **4** obtained from THF/MeOH, and (**c**) experimental of **4** obtained from THF/EtOH.

.


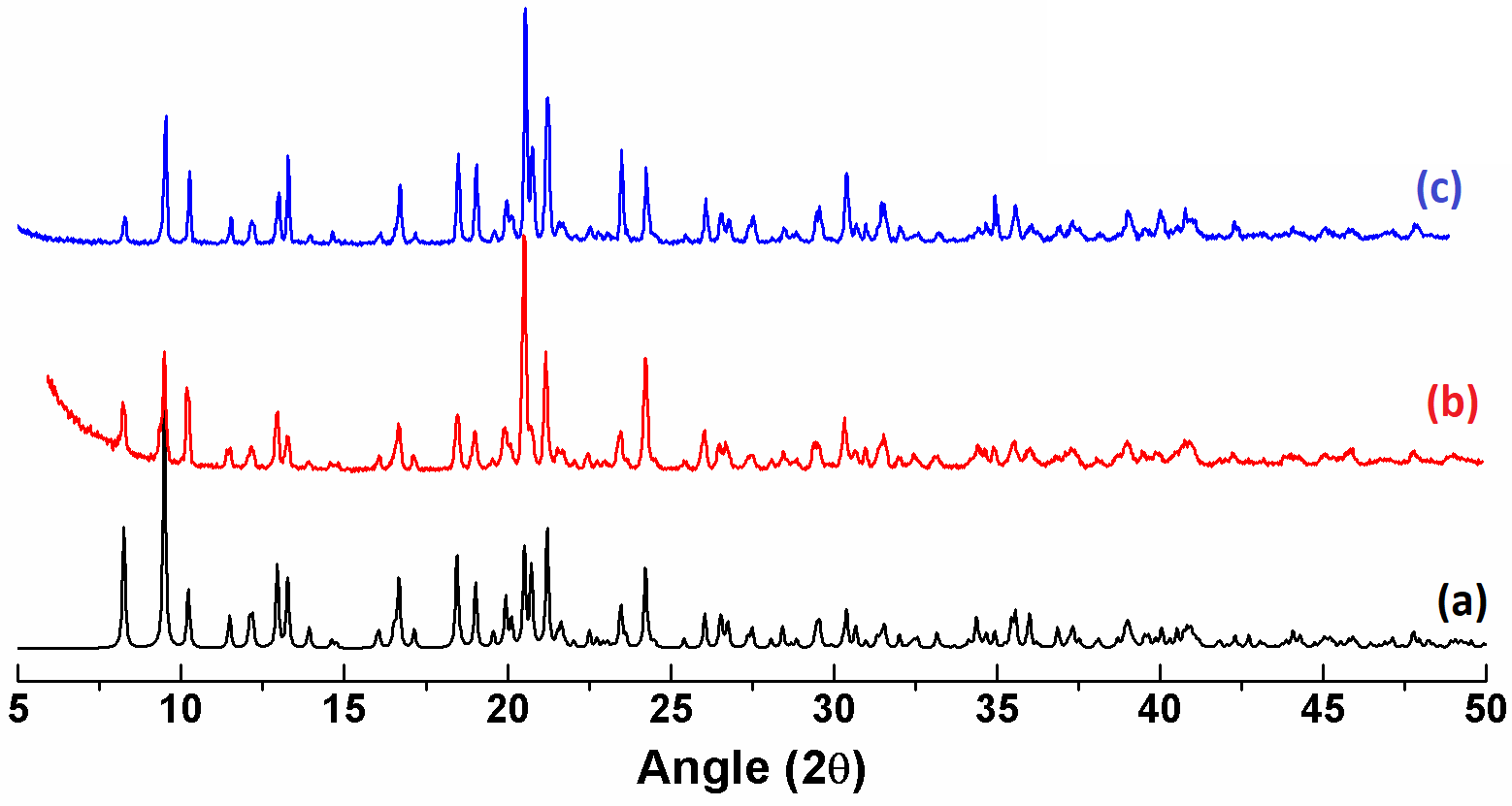


**Figure S5.** Powder X-ray patterns of **2.** (**a**) Simulation, (**b**) experimental, and (**c**) in MeOH after one month.


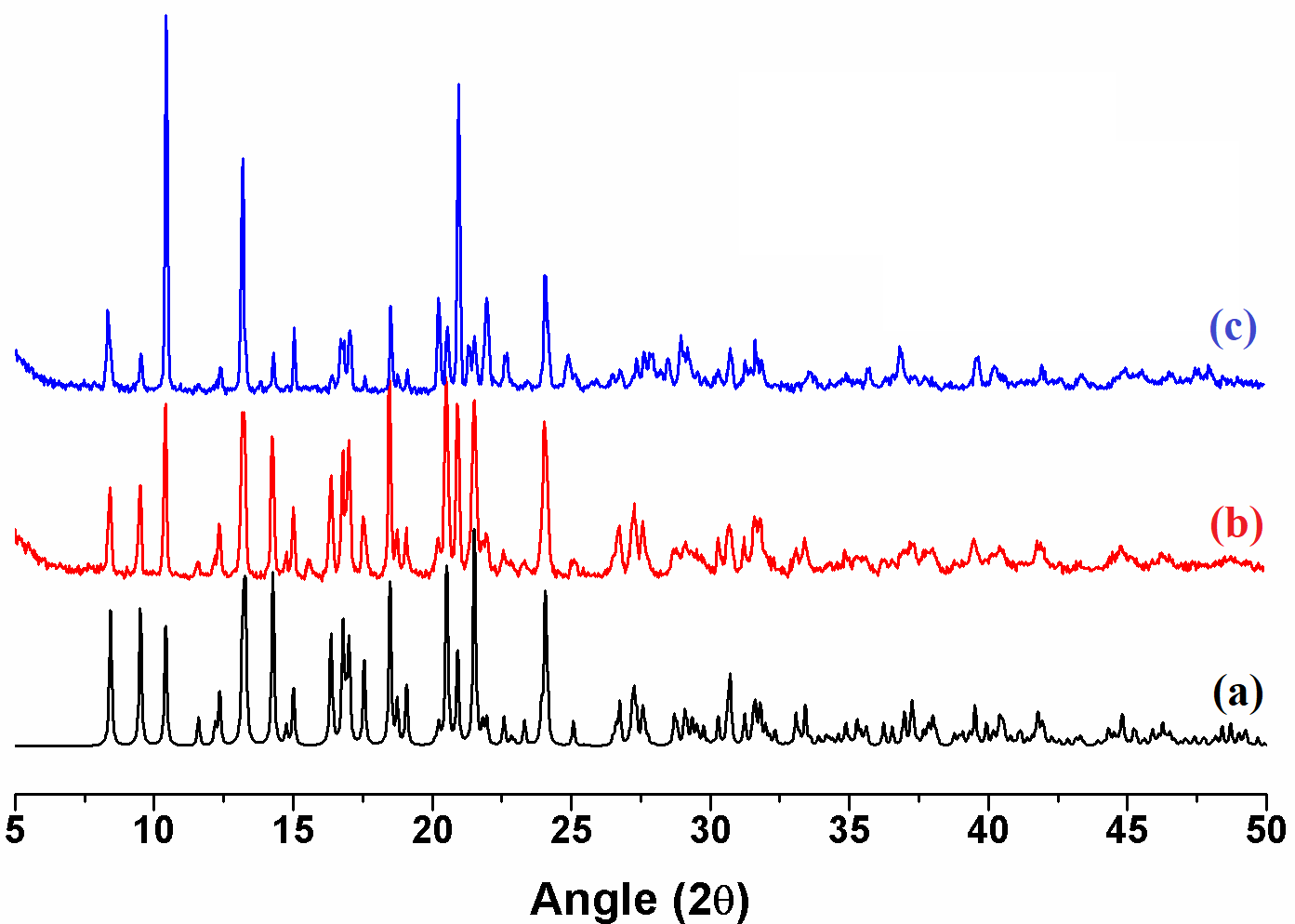


**Figure S6.** Powder X-ray patterns of **3**. (**a**) Simulation, (**b**) experimental, and (**c**) in MeOH after one month.


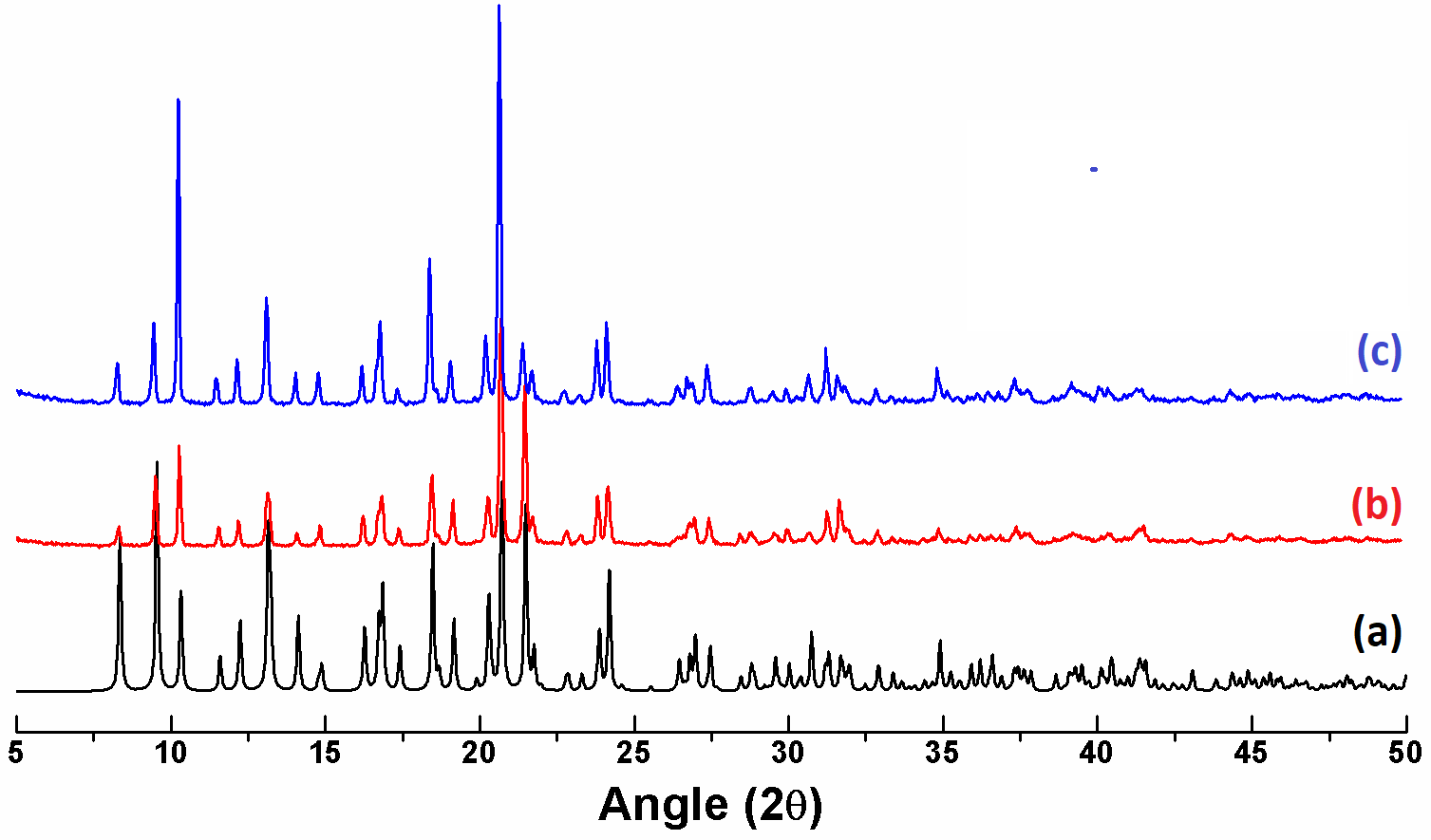


**Figure S7.** Powder X-ray patterns of **3**. (**a**) Simulation, (**b**) experimental, and (**c**) in solvothermal reaction with MeOH.





**Figure S8.** Powder X-ray patterns of **4**. (**a**) Simulation, (**b**) experimental, and (**c**) in MeOH after one month.


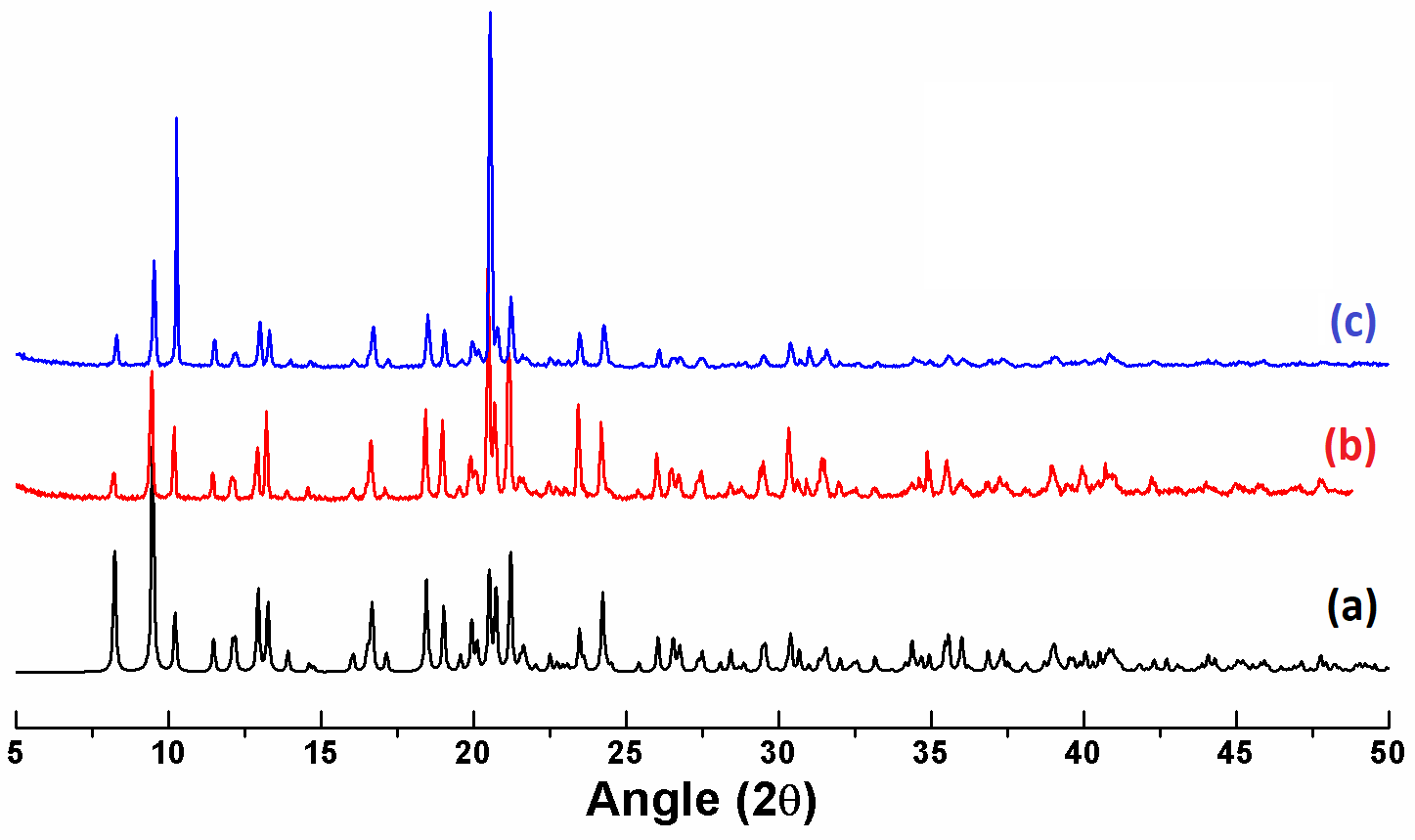


**Figure S9.** Powder X-ray patterns of **4**. (**a**) Simulation, (**b**) experimental, and (**c**) in solvothermal reaction with MeOH.

**

**
